# Long-Term Effects of the Individual Placement and Support Intervention on Employment Status: 6-Year Follow-Up of a Randomized Controlled Trial

**DOI:** 10.3389/fpsyt.2021.709732

**Published:** 2021-10-12

**Authors:** Eva-Maria Pichler, Niklaus Stulz, Lea Wyder, Simone Heim, Birgit Watzke, Wolfram Kawohl

**Affiliations:** ^1^Department of Psychiatry, Psychotherapy and Psychosomatics, University of Zurich, Zurich, Switzerland; ^2^Psychiatric Services Aargau, Windisch, Switzerland; ^3^Integrated Psychiatric Services Winterthur-Zurcher Unterland (IPW), Winterthur, Switzerland; ^4^Praxis Dr. Pramstaller, Uetikon am See, Switzerland; ^5^Clinical Psychology and Psychotherapy, Department of Psychology, University of Zurich, Zurich, Switzerland; ^6^Clienia Schlössli AG, Oetwil am See, Switzerland

**Keywords:** individual placement and support (IPS), supported employment (SE), vocational rehabilitation (VR), serious mental disease, social security disability insurance, job sustainability

## Abstract

People with mental illness often experience difficulties with reintegration into the workplace, although employment is known to assist these individuals in their recovery process. Traditional approaches of “first train, then place” have been recently replaced by supported employment (SE) methods that carry strategy of “first place, then train.” Individual placement and support (IPS) is one of the best-studied methods of SE, which core principles are individualized assistance in rapid job search with consequent placement in a paid employment position. A considerable amount of high-quality evidence supported the superiority of IPS over conventional methods in providing improved employment rates, longer job tenure, as well as higher salaries in competitive job markets. Nonetheless, our knowledge about the IPS-mediated long-term effects is limited. This non-interventional follow-up study of a previously published randomized controlled trial (RCT) called ZhEPP aimed to understand the long-term impact of IPS after 6 years since the initial intervention. Participants from the ZhEPP trial, where 250 disability pensioners with mental illnesses were randomized into either IPS intervention group or treatment as usual group (TAU), were invited to face-to-face interviews, during which employment status, job tenure, workload, and salaries were assessed. One hundred and fourteen individuals agreed to participate in this follow-up study. Although during the first 2 years post-intervention, the IPS group had higher employment rates (40% (IPS) vs. 28% (TAU), *p* < 0.05 at 24 months), these differences disappeared by the time of follow-up assessments (72 months). The results indicated no substantial differences in primary outcome measures between IPS and TAU groups: employment rate (36 vs. 33%), workload (10.57 vs. 10.07 h per week), job tenure (29 vs. 28 months), and salary (20.21CHF vs. 25.02 CHF). These findings provide important insights regarding the long-term effects of IPS among individuals with mental health illnesses. Further research is required to advance the current knowledge about IPS intervention and its years-long impact.

## Introduction

Problems with mental health may cause an enormous burden to affected people, their beloved ones, and society in general. According to the Global Burden of Disease Study, 19% of all years lived with disability are caused by mental and addictive disorders affecting over 1 billion people worldwide as of 2016 (1). Mental illness can be disabling and diminish the ability to work in the competitive employment market. Poor health and diminished productivity due to mental problems amount to 3% of Swiss GDP and could lead to $6 trillion in annual loss globally (2, 3). Once work has been suspended, reintegration may be difficult and lead to long-term unemployment and chronic disability.

Nonetheless, the reintroduction of people with mental illness into the workforce holds medical, personal, and economic benefits both from an individual and societal perspectives (4–8). More recent evidence supports the assumption that employment benefits prevail for people with and without mental illness (8–11). Conversely, the negative effects of unemployment may be particularly profound for people with mental illness (12, 13).

Different approaches exist for the rehabilitation and reintegration of people with mental illness into the employment market. Following the pre-vocational rehabilitation (PVR) approach, the first step of reintegration involves training in protected work environments to instill habits that align with employer expectations and increase chances of successful integration into the competitive employment market (14). This stepwise approach is called “first train, then place” assuming that stabilization is a prerequisite to successful reintegration (15). However, high-level work overload as well as inability to integrate into the competitive job market limited the applicability of PVR, leading to stigmatization and social isolation of affected persons (16).

Following changes in the perception of people with mental illness and their ability to reintegrate, novel alternative rehabilitation approaches have begun to emerge, including the model of supported employment (SE). The latter aims to support affected individuals in seeking employment in the competitive market and receive coaching during their initial employment phase; thus, having a “first place, then train” approach (17). The superiority of the SE model over the PVR approach has been confirmed by a large number of studies (18, 19).

The individual placement and support (IPS) model represents the best described SE approach and has been developed based on empirical data, following defined principles and targets (18, 19). These include direct and rapid employment in the competitive market, under consideration of patient preferences, with unlimited duration of individual coaching in a non-institutional environment. Reintegration into the employment market and building a social network is the central aspect of patient management, integrating work and therapy, which is led by an interdisciplinary team (18, 19). A considerable amount of high-quality evidence from randomized controlled trials (RCT) supports the efficacy of this approach in providing improved employment, which has been validated in a number of countries and slowly becoming a standard of practice (18, 20–24). As such, aside from higher income and improved employment status, IPS was demonstrated to lead to enhanced job tenure, which is a key employment outcome for unemployed populations (18, 25–27).

Individuals who had undergone IPS show substantially high job tenure rates (i.e., the duration of the longest-held competitive employment job) ranging from as short as 7 weeks up to over 51 weeks in studies with a follow-up period of 12 to 24 months (27–36). This considerable variation in job tenure rates refers to distinct study populations and their specific characteristics (e.g., age, type of mental illness, level of education) (18, 37). The most frequently implemented strategies to address issues of job tenure among IPS service users include work-related skills training (e.g., social competencies, general skills) (28, 29). Nonetheless, cognitive training (e.g., cognitive remediation or errorless learning), psychological interventions, and supported education have also been described as potential tools of improvement (38–41). Moreover, support from peers, family members, or friends may enhance the effects of IPS on job tenure. In contrast, mobile apps to support task management, self-management programs, workplace accommodations, and working alliance between employee and employer can also be promising adjustments (42).

Nonetheless, the data on the long-term impact of IPS is still lacking. The latter is of particular concern, as one of the main challenges for work reintegration is the employment sustainability among people with mental disorder since it tends to be lower compared to individuals that were unemployed due to other reasons (43). Thus, there is a need to assess the durability of the positive effects of the intervention.

Another important aspect of IPS intervention is fidelity, which refers to the quality and success of the program implementation. Studies showed that higher IPS fidelity was associated with higher job tenures and increased employment rates (44, 45).

The return-to-work is hindered by important individual factors, such as somatic disorder, older age, higher levels of impairment, limited activity, increased work demands (46–48), as well as external barriers, such as stigmatization, unavailability of suitable jobs, lack of social support, and absence of financial incentives or subsidies for the promotion of integration (3, 48, 49). Poor information and knowledge about employment benefits and opportunities among healthcare providers and affected people can also contribute to the existing problem (3).

Switzerland provides people who are unfit to work due to injury or disease with a partial (25% of the total) or full (75% from the total) invalidity pension (IV, Invalidenversicherung), which is currently drawn by 4% of the total population. Nearly half of these pensions (49%) are provided to people with mental illness. Since 2012, incentives are offered to reintegrate IV beneficiaries into the employment market, following the PVR and IPS approach (50).

The present work is a follow-up study of the previous randomized controlled trial (RCT), which demonstrated higher efficacy of IPS compared to the standard approach during 46-months of the study period in achieving competitive employment acquisition among Swiss disability pensioners with mental illness (32 vs. 12%, *p* < 0.0001) (51). Thus, this study aimed to investigate the long-term effects of IPS intervention on job tenure and the factors influencing it by performing follow-up interviews of participants from the ZhEPP trial 6 years since the start of the original intervention.

## Methods

### Study Design and Participants

This study is an observational follow-up of the previously published RCT (51). The Zurich Integration Pilot Project (ZhEPP, *German*: Zürcher Eingliederungs Pilot Projekt) was conducted between January 2011 and September 2014 in order to investigate the efficacy of the IPS approach in reintegrating pensioners with mental illness into the competitive employment market (52). The ZhEPP was a collaboration between the Psychiatric University Hospital of Zürich (PUK, Psychiatrische Universitätsklinik) and the IV authority in Zürich. It was carried out at the PUK among 250 newly assigned IV beneficiaries who were disability pensioners with mental health problems. Individuals of working age, who had been IV beneficiaries for a maximum of 1 year due to a mental illness, and wished to return to regular employment, were included. Those with intellectual disabilities and organic mental disorders were excluded. The enrolled participants were randomized to either IPS (intervention group) or treatment as usual (TAU or control) group. Upon allocation, participants were interviewed every 6 months over the course of 2 years, with a total of five interviews regarding their employment status and additional job-related parameters. Each interview involved face-to-face conversations conducted by the research team and took half a day for each participant. Further details can be found in the published study protocol (52).

Our follow-up was conducted from November 2017 to February 2018 and did not involve any further interventions except for single timepoint interviews aiming to assess the long-term impact of IPS. Two psychologists of our research team carried out face-to-face interviews, identical to the ones from the original RCT. Only a single interview was conducted per participant during the follow-up period.

### Procedures

#### Intervention and Control Group (Original ZhEPP-RCT)

During the preceding RCT, participants in the intervention group received free job coaching from one of the four psychologists of the research team (51). Job coaches followed the IPS principles and were requested to support patients during their job search and employment following patient preferences, emphasizing patient independence. They also provided assistance and support during the application process, during actual employment, as well as when a participant lost the job. Participants from the TAU group were free to choose other vocational services aimed to improve employment status; however, they were not supported by a job coach during the period of the original RCT. Implementation of the IPS approach was assessed every 3 months by an amended version of the 15-point fidelity scale (53). After the end of the ZhEPP trial, participants had an option to continue using IPS and TAU services, respectively, voluntarily.

#### Follow-Up Interviews

The follow-up interviews took place between November 2017 and February 2018 and are to be regarded as a non-interventional randomized controlled study. All original participants were contacted by phone and email. Those who could not be reached via these two means of contact were also asked to participate again by post, in which questionnaires were delivered and received in an enclosed envelope free of charge. For most participants, interview appointments lasting about half a day were set upon consent to participate. The effort of each participant in the follow-up interviews was reimbursed with a shopping voucher worth CHF 50 (~$55). The primary outcome for the follow-up interview was assessing job sustainability for IPS compared to TAU based on the employment status 6 years post-intervention. The secondary outcome was the long-term impact of two approaches on job tenure and salary by assessing study participants' employment duration and wages.

#### Material

Questionnaires for the ZhEPP were modeled based on the multicenter EQOLISE study (54). Several sections from the original trial were used for this study. For the employment assessment, Job Status Questionnaire was used, in which participants were requested to indicate whether they gained employment through their effort (1), the IPS coach (2), or other means (3). Participants could also answer in an open response format (i.e., using their own words) about the current employer, the job, the start of the job, and the reason for possible interruptions in employment. Follow-up questions included information about current employers, project details, the start of employment, and potential breaks in employment. For the follow-up interviews, the questionnaire used was amended to fit the aim of the study. Participants were asked whether they were employed in the first (competitive) or second (protected) employment market, were in training, unemployed, or retired. The protected type of employment market lacks competition among applicants and employees and is characterized by a state-subsidized nature of relationships, which are usually not remunerated at market rates. If employed, further requested information included employer details, nature of work, working hours, and weekly salary. Participants were asked to indicate the number of vacancies (i.e., acquired job positions) and months worked for the year prior to the interviews. Regarding the period of 6 years, specific questions were only addressed about the number of positions acquired and months worked in the primary labor market (i.e., not in training).

The IPS fidelity scale was also used to evaluate the efficacy of IPS implementation on employment status by measuring the adherence to the core principles of this approach (55).

Patient demographics were recorded using the Client Sociodemographic and Service Receipt Inventory (CSSRI-EU), which was translated into German, and a user manual with definitions and instructions for understanding was provided (56). The questionnaire was divided into five areas. In the part of socio-demographic information, the variables age, gender, marital status, school education, and vocational training are recorded. In the section on the living situation, questions addressed the lifestyle and housing, as well as possible changes during the observation period. In the third part, the employment status, the occupation, the days of work absence, and the type and amount of social support benefits were recorded. In the fourth section, for the use of care services, information on possible inpatient, partial inpatient, outpatient, and complementary care services, as well as contact with the police and the judiciary, was collected. The fifth section on medication was used to document the type, name, dosage, and frequency of the medication taken. The third section on the employment situation was shortened, as the information could already be collected through the job status questionnaire.

#### Data Analysis

Data analysis was performed using IBM SPSS version 24 (Armonk, NY, USA). The normal distribution of the variables was examined visually by histograms and Q-Q-plots and confirmed by Shapiro-Wilk tests. Differences in-between groups on continuous variables were analyzed by *t*-tests for independent samples. In the case of the non-normal distribution of a variable, analysis was performed using the Mann-Whitney-U test. In-between group differences of categorical variables were examined using the Pearson Chi-squared test. In the case of expected cell frequencies of <5, variables were analyzed using Fisher's exact test. A *p*-value of ≤ 0.05 was considered statistically significant.

Longitudinal inter-group differences were analyzed using a generalized estimated equation (GEE) model, which is perfectly suited for examining dichotomous data over time, taking into account dependencies of repeated measurements of a participant and the time course in the model (57). GEE was squared to allow more flexibility in handling possible fluctuations regarding the primary outcome during different measurement points.

For identification of factors influencing the effects of job coaching on employment status in the competitive market, only participants in the intervention group (*n* = 127) were analyzed by means of logistic regression models (Supplementary Table 1) (58). As the selection of potential predictors of employment in the competitive market was based on theoretical assumptions, the inclusion method was used.

### Ethical Considerations

The ZhEPP and the follow-up study were approved by the local ethics committee (approval number *2016-01636*) and were carried out in line with the Helsinki declaration. Data confidentiality was ensured by providing each participant with a unique participant ID by the interviewers, which was henceforth used for data collection and analysis of both ZhEPP and the follow-up study.

## Results

### Representativeness

The representativeness of the study population was counted from the start of the trial to the end of the follow-up period considering the present work as a continuation of the ZhEPP. Out of the 250 participants enrolled in the original ZhEPP study, two did not show up for the first appointment and were excluded from the baseline assessment, reducing the original sample size of the ZhEPP to 248 (36). For the follow-up interviews, 25 out of the 248 participants (10.1%) could not be contacted due to missing valid contact information. Further, 68 participants (27.4%) were excluded as they could not be reached or did not respond. Twenty-four participants (9.7%) had no interest in participating in the study, 15 people (6%) decided against participation after receiving the questionnaire, and 3 people (1.2%) had died. In total, 114 people (46% of the baseline sample) participated in the follow-up interviews. The difference in dropouts between the study groups (18% (IPS) vs. 14% (TAU)) was not significant (data not shown).

Most of the participants were lost in the first part of the ZhEPP trial (16%, *n* = 40). In order to assess representativeness, we compared the data set (follow-up time point, *n* = 114) to those participants of ZhEPP who did not take part in the follow-up interviews (*n* = 134). Comparison of the data sets reveals a significantly higher percentage of people with the affective disorder as the primary diagnosis (χ^2^(1) = 7.73, *p* = 0.005, Cramers *V* = 0.189), as well as a significantly lower percentage of people with the primary school as their highest level of education (χ^2^(1) = 4.04, *p* = 0.04, Cramers *V* = −0.138) in the group evaluated in the follow-up study, compared to those who did not participate. No further differences were noticed (see Supplementary Table 2). The analysis was not controlled for the dropout time or the number of completed questionnaires pursuing the intention-to-treat analysis approach, which most closely corresponds to clinical practice.

### Patient Demographics

A comparison of the patient demographics of the IPS coaching group to the TAU group revealed significant differences between the groups regarding primary school rates. In the IPS coaching group, significantly more attendees reported having only a primary school degree than TAU controls (*p* = 0.04, Cramers *V* = 0.204) (Table 1).

**Table 1:** Participant characteristics Follow-up study (*n* = 114).

	**IPS coaching** ***n* = 62**	**TAU control *n* = 52**	***p*-value**	**Total**
Women^a^^d^	32 (51.6%)	30 (54.4%)	0.516	62 (54.4%)
Age, yrs at follow-up time point (*M±SD*)^c^	47.32 ±10.44	50.68 ±10.62	0.953	48.75 ±10.59
Number of years between first contact with psychological care and begin of study^a^^c^ (*M±SD*)	11.35 ±9.28	10.61 ±7.75	0.124	11.03 ±8.61
**Clinical diagnosis^a^^d^**				
Affective disorder	36 (58.1%)	30 (58.8%)	0.984	66 (58.9%)
Schizophrenia, schizoaffective disorder	8 (13.1%)	3 (5.9%)	0.200	11 (9.8%)
Personality disorder	7 (11.5%)	8 (15.7%)	0.515	15 (13.4%)
Other	10 (16.4%)	10 (19.6%)	0.658	20 (17.9%)
Comorbidities (yes)^a^^d^	26 (41.9%)	25 (48.1%)	0.511	51 (44.7%)
**Hospitalizations at follow-up time point^a^^d^**				
None	20 (37%)	17 (41.5%)	0.661	37 (38.9%)
1–5	28 (51.9%)	19 (36.5%)	0.591	47 (49.5%)
6–10	4 (7.4%)	5 (12.2%)	0.430	9 (9.5%)
11+	2 (3.2%)	0	0.213	2 (2.1%)
**Highest Level of Education at follow-up time point^b^**				
Primary School	33 (56.9%)	16 (35.5%)	0.040	49 (47.6%)
Secondary school diploma	8 (13.8%)	12 (26.7%)	0.089	20 (19.4%)
Another diploma	17 (29.3%)	16 (35.6%)	0.415	33 (32.0%)
**Living situation at follow-up time point^b^^d^**				
Single	34 (58.6%)	21 (47.7%)	0.274	55 (53.9%)
Living with partner/married	17 (29.3%)	19 (43.2%)	0.147	36 (35.3%)
Living with relatives	4 (6.9%)	4 (9.1%)	0.723	8 (7.8%)
Living with others	3 (5.2%)	0	0.257	3 (2.9%)

*IPS, Individual Placement and Support; M, Mean; SD, standard deviation; TAU, treatment as usual. Data correlate with number (n) per group (valid percentage)*.

a *In relation to baseline*.

b *Smaller sample size due to lack of obtained information*.

c *Mann-Whitney-U-Test*.

d *Chi-Square Test using Fisher's exact test if cells had expected count <5*.

*p > 0.05, no significant differences between the groups*.

### Employment Status

Out of the 114 participants of the study at a follow-up time point, 36 (31%) were not in employment, 52 (45.1%) were employed in the competitive (primary) job market, and 21 (18.6%) held a position in the protected (secondary) job market. Furthermore, 5 participants (4.4%) had retired (see Table 2). No significant differences were observed between the IPS coaching group and the TAU group.

**Table 2:** Employment status at the follow-up interviews.

	**IPS Coaching** ***n* = 62**	**TAU Control** ***n* = 52**	***p*-value**	**Total**
Not employed	21 (33.9%)	15 (28.8%)	0.565	36 (31.9%)
Employed primary job market	27 (43.5%)	25 (48.1%)	0.629	52 (45.6%)
Employed secondary job market	12 (19.4%)	9 (17.3%)	0.779	21 (18.4%)
Retired	2 (3.2%)	3 (5.8%)	0.658	5 (4.4%)

*Primary job market refers to employment in the competitive job market, secondary job market refers to protected employment*.

*IPS, Individual Placement and Support; TAU, treatment as usual*.

*Chi-Square test using Fisher's exact test if cells had expected count <5. P > 0.05, no significant differences between the groups*.

### Employment Duration and Wages

Further assessment of the employment situations and the employment history was conducted in order to evaluate differences in the quality of employment between the groups. Comparison of the number of acquired positions and the employment duration during the last 6 years revealed no differences between the study groups (see Tables 3, 4). Participants with competitive and protected jobs in the IPS coaching group worked on average 29 months, whereas those in the TAU group 28 months, with 1.17 and 1.23, acquired positions over the study duration, respectively. The workload was also comparable between the groups, with participants in the IPS coaching group working an average of 16.60 h per week in the competitive job market, compared to 17.31 h per week in the TAU group. Hourly At follow-up time points, hourly wages (for both types of jobs) at follow-up time points did not differ significantly between the groups (Table 5). Likewise, the analysis of employment rates at the selected follow-up time point showed no differences between TAU and the IPS approach (Table 6).

**Table 3:** Model estimate of the number of positions acquired in the primary work market over the various time points.

	**Constant**	**SE**	***p*-value**	**Exp (*B*)**
Intercepts	−1.105	0.2118	0.000^***^	0.331
Group	0.116	0.288	0.688	1.123
Time	−0.091	0.1025	0.373	0.913
Time 2	0.034	0.0195	0.081	1.035
Group Time	0.571	0.1453	0.000^***^	1.770
Group Time 2	−0.114	0.0269	0.000^***^	0.892

*Group, IPS coaching group and control group; Time, six different time points; SE, standard error; Exp (B), effect size; df, 1; Goodness for fit (QIC) = 1857.096*.

*** *p < 0.001*.

**Table 4:** Positions and employment duration in the follow-up study.

	**IPS coaching group** **(*n* = 62)**	**TAU group** **(*n* = 52)**	**Total**	***p*-value**	**Effect size**
Acquired positions over the last 6 years (*M±SD*)^a^	1.23 ± 1.33	1.1 ± 1.31	1.17 ± 1.32	0.261	−0.098
Months employed during the last 6 years (*M±SD*)^a, b^	28.78 ± 29.44	27.90 ± 28.67	28.38 ± 28.97	0.386	0.157

*IPS, Individual Placement and Support; M, Mean; SD, standard deviation; TAU, treatment as usual*.

a *Mann-Whitney-U-Test, one-tailed*,

b *Smaller sample size due to lack of obtained information*.

*p > 0.05, no significant differences between the groups*.

**Table 5:** Workload and salary in the follow-up settings.

**All employed participants**	**Total (*n* = 73)^c^**	**IPS (*n* = 39)^c^**	**TAU (*n* = 34)^c^**	***p*-value**	**Cohens d**
Workload (Hours/week)^a^	10.57 ±12.48	10.07 ±11.88	11.15 ±13.25	0.443	0.086
<10 h^b^	21 (28.76%)	12 (30.77%)	9 (26.47%)		
10–30 h^b^	0.40 (54.79%)	23 (58.98%)	17 (50%)		
>30 h^b^	8 (10.96%)	3 (7.69%)	5 (14.71%)		
Wages in CHF (±SD)^a^	22.30 ±13.28	20.21 ±13.22	25.02 ±13.12	0.101	0.365
**Primary job market only**	**Total (*****n*** **=** **52)**^c^	**IPS (*****n*** **=** **27)**^c^	**TAU (*****n*** **=** **25)**^c^	***p*** **-value**	**Cohens d**
Workload (Hours/week)^a^	15.73 ± 13.26	16.60 ± 12.84	17.31 ± 13.41	0.451	0.054
<10 h^b^	19 (36.5%)	11 (40.7%)	8 (32%)		
10–30 h^b^	25 (48.1%)	13 (48.1%)	12 (48%)		
>30 h^b^	7 (13.5%)	3 (11.1%)	4 (16%)		
Wages in CHF (±SD)^a^	25.98 ± 11.91	25.02 ± 11.39	27.06 ± 12.64	0.308	0.17

*CHF, Swiss Francs; IPS, individual Placement and Support; SD, standard deviation; TAU, treatment as usual*.

a *Marry-Whitney U-test, one-sided;*

b *Pearson Chi-squared test, one-sided;*

c *small sample size due to missing values*.

*p > 0.05: no significant differences between study groups were founded*.

**Table 6:** Employment rate across the time points (*n* = 248).

**IPS Coaching Group**			**TAU Group**			
**TP**	**Number**	**% in M1^b^**	**Number**	**% in M1^b^**	***p*-value**	**Cramers *V***
0	34	26.8	30	24.8	0.833	0.023
1	46	36.2	29	24.0	0.05^*^	0.133
2	52	40.9	29	24.0	0.007^**^	0.181
3	56	44.1	31	25.6	0.004^**^	0.194
4	51	40.2	34	28.1	0.062^*^	0.127
Follow-up^a^	46	36.2	40	33.1	0.595	0.041

*IPS, Individual Placement and Support; M1, primary job market; TAU, treatment as usual; TP, time point. Analysis based on intention-to-treat, LOCF*.

a *For participants retired at follow up time point, the values obtained at time point 4 were carried over*.

b *Valid percentage indicated*.

* *p < 0.05*,

** *p < 0.01. Pearson Chi-squared test, two-tailed, with Yates correction (continuity correction), df = 1*.

## Discussion

### Main Findings

Our six-year observations from the ZhEPP-RCT data indicated that both IPS and traditional approaches effectively provide competitive job positions for individuals with mental illnesses in Switzerland. However, the positive effects of IPS implementation on employment seem to decrease over time. The difference in the effects from the IPS and TAU groups were prominent after the first 6 months post-intervention (36 vs. 24% in employment, respectively). The percentage of participants who received IPS with the protective or competitive type of job increased from the starting 26 to 44% by the second year, whereas no major changes were observed in the control group (25 to 26%, respectively). Nonetheless, the follow-up interviews conducted 6 years after the initial interventions demonstrated that the gap between the study groups diminished over the years and was not significant anymore due to a decrease in the employment rate in the IPS group. Only 36% of participants of the IPS group still held a job in either competitive or protected type of employment, while the TAU group had 33% employed participants. Comparison of the employment rates in the competitive job market at follow-up time point revealed no significant difference between the study groups. Nevertheless, at the end of the 6-year follow-up, the employment rates were significantly higher than the study's baseline. These findings provide important insights regarding the long-term effects of distinct intervention strategies aiming at personal and/or clinical rehabilitation from mental illness.

Investigation of workload and remuneration also revealed no difference between the IPS coaching group and the TAU group. Approximately half of the participants employed in the competitive market worked part-time between 10 and 30 h. In comparison, a third worked <10 h, primarily corresponding to the preferences of a participant and the preferences of the job coach (received at the beginning of the coaching during the original RCT). The latter can potentially be a reason for longer job duration compared to other studies. However, there was a comparable distribution of employment in the primary and secondary markets in both groups. The average salary of participants of the IPS coaching group did not exceed the average income of the TAU group, again contradicting the findings from previous studies. One of the potential explanations of these findings could be the issue that the interventions (i.e., IPS and TAU) from the original ZhEPP study were not carried out continuously in all participants after the end of the trial. Since study groups showed similar results in primary endpoints (i.e., job tenure, employment rates, and workload), one may argue that the superiority of IPS over conventional methods tends to wean over the years, especially if not continuously supported. Nonetheless, it is important to keep in mind that a range of various factors could potentially impact employment outcomes. Thus, generalizability and interpretation of the presented findings require vigilance and careful consideration of the context and details of settings.

### Comparison With Findings From Previous Studies

Implementation of the IPS leads to higher rates of employment and job tenure and improved salary (18, 23). The overwhelming amount of evidence supported the superiority of IPS compared to traditional methods of interventions changing the standards of practice in many countries (23, 59–62). For example, in the Netherlands, the number of individuals involved in IPS programs doubled from 2016 to 2017 primarily due to national funding of such services (63).

The findings from our study are partially comparable to and somewhat distinguished from the previously published literature results. The employment outcomes (i.e., job tenure and employment rates) may range depending on participants' study characteristics and individual features and applied interventions (64). For example, an RCT of 162 participants with schizophrenia receiving IPS showed that 50% of employment rate with 25.47 weeks in job tenure after 1 year of follow-up (65). Throughout the 18-month follow-up period, IPS-supported employment led to 68.6% of job acquisition among 541 unemployed US-veterans with post-traumatic stress disorder in a multi-site RCT (66). Meanwhile, in an RCT of 85 participants with severe mental illness and justice involvement, 31% of people who received IPS acquired a competitive type of job compared to only 7% in the control group at 1-year of follow-up (*p* < 0.01) (67). The trends observed in the first two years after intervention (as described in the ZhEPP trial) (51) are similar to the ones described in the literature (68–70), in which the between-group differences became significant after a half of year of the intervention and may continue to rise or stay stable up to 1 or 2 years (27, 71, 72). As in our study, the likelihood of acquiring a job position diminishes over time among individuals with IPS, despite overall higher employment rates (73). These findings may serve as an important clue for practitioners regarding recovery and rehabilitation planning and outcome anticipation for patients and clinicians (68).

The data on the long-term impact of IPS is, however, limited. Among 151 individuals with severe mental diseases demonstrated higher employment rates in those receiving IPS strategy compared to people managed with the traditional methods (44 vs. 25%) were found in a 30-month multi-site RCT from the Netherlands (74). A study of 95 persons with mental disorders from Italy demonstrated steadily rising rates in competitive employment for almost up to 4 years of follow-up, claiming the sustainability of IPS effects (75). In contrast to our data, 41% of participants of this study had a competitive type of job by the fourth year of observations (75). Our results also contradict the findings from a study with a similar methodological design by Hoffmann et al., in which 100 Swiss residents with mental disorders receiving IPS had higher rates of competitive employment 5-years after the intervention compared to those with conventional approach (65.2 vs 33.3%, *p* < 0.002) (76). The advantage of IPS in various employment outcomes was similar during the first 2 years of the follow-up and remained significant afterwards, indicating that the positive and sustainable effects of IPS on work over the 5-year follow-up period (76).

**Figure 1:**
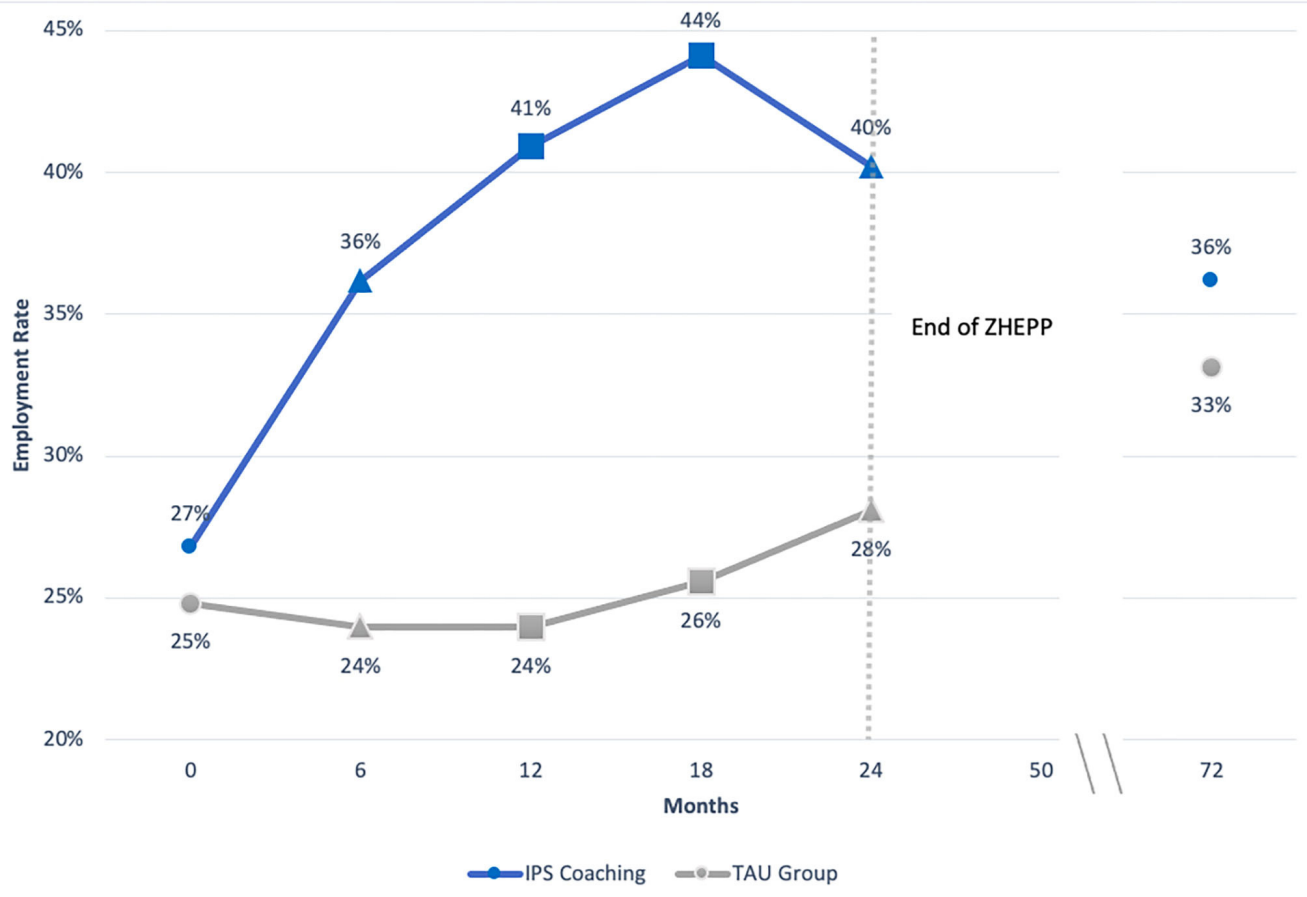
Employment rate in ZhEPP and its follow-up based on *intention-to-treat*, LOCF analysis. IPS=Individual Placement and Support, M1 = primary job market, TAU = treatment as usual. For participants retired at follow-up time point (72 months after start of study), the values obtained at time point 4 were carried over.

One of the factors possibly explaining the differences of our study findings is the fact that most of the participants reported the initial time point of seeking psychological care on average as 10 years ago, even though all of the participants had qualified to obtain IV pension within the last year before the beginning of the study. This indicates that many of the survey participants had been affected by their disorder long-term. Furthermore, we found a significantly higher proportion of participants from the IPS group having primary school as the highest academic degree, which could have impacted the outcomes. The observed assimilation in the employment rate of the IPS coaching and the TAU group at the follow-up time point may be based on changes in legislation during the study period. In 2012, a revision of measures to support the reintegration of people with disabilities came into effect, providing increased support during the job search. Therefore, participants of the TAU group may have benefitted from this revision, which may have caused a steady increase in the employment rate observed in the TAU group over time. It was beyond the scope of this study to investigate the impact of the revised legislation on the results. Nonetheless, the IPS strategy resulted in a continuous level of employment of 36% (Figure 1) of participants in the competitive job market across study duration, thereby showing similar results to other long-term studies (76).

In addition, ethical considerations could not prevent participants of the TAU group from joining other support programs. Participants of the TAU group were free to use other services for employment acquisition. However, this data was not recorded and therefore not considered in the analysis, possibly masking a more pronounced difference between the groups.

### Barriers and Facilitators of Successful IPS Implementation and Employment

Aside from methodological differences, the distinction in employment sustainability as well as in the magnitude of the effects among the study results may correspond to several important factors that either enhance the impact of an intervention or hinder it. Multiple studies cited lack of disability benefits cited lack of disability benefits cited lack of disability benefits as a key environmental barrier for employment (49).

Another critical obstacle for people with mental disorders is motivation to acquire a job, which refers to practical steps and strategic efforts required for potential employment (49). Individual perception of multiple impediments can lead to hesitance and inaction, which can be combated with the person's empowerment (39). Individualized support is perceived by people with mental illnesses seeking employment as an important facilitating factor, which is a challenging task for social workers and clinicians involved in IPS programs (77, 78). The type of mental disorder can also affect program's efficacy for different outcomes (21). External emotional support from peers and family members is critical for rehabilitation and recovery (42, 79). Cognitive and psychological interventions along with supported education were shown to be important contributors to enhancing employment status (42, 80).

The relationship between the employer and an employee with mental illness can play a crucial role, which can depend on the individual internal barriers of the person (e.g., disease nature, severity of impairment), as well as competencies of the employment specialist (34). Therefore, not only people with mental disabilities should receive training to increase job tenure and employment sustainability, but also employers to help to solve the common problem (81–83).

Type of the mental issue is also critical as it seems to be more effective in some disorders and less and in others (21, 84). A systematic review of studies assessing IPS efficacy in individuals with substance use disorders found that episodic treatment of the disease and risk of relapse of the mental disease were among important barriers in implementing IPS (22). Often, the ethical part of the mental disease is not considered. Qualitative analysis by de Greef showed that the risk of disclosure of the medical diagnosis might represent a potential issue for people with mental conditions seeking employment (85). Lastly, since the spread of the IPS model, different variants and types were invented to fit the needs of specific population groups (50, 86, 87), explaining the individualistic approach to the implementation of this strategy and an opportunity for further development improvements and developments (88).

### Limitations and Strengths

The results of this study should be interpreted carefully, as our study has several important limitations. One of the key limitations is the differences in the level of education between study groups. Significantly more participants of the IPS group had primary school as the highest achieved academic level, compared to the controls, which could be reflected in their qualification and remuneration. This could have further affected the long-term outcomes of the duration of employment, workload, or salary for the IPS group, which did not differ from the TAU group. Furthermore, we could not provide longitudinal associations between interventions and outcomes; thus, limiting the interpretation of trends because of interruption in the timeline. The number of people with affective disorders was slightly higher in the participants of the follow-up survey (58.9%) than in those who did not participate (40%), the impact of which on main outcomes cannot be ruled out completely. Recall bias is another potential limitation of the presented work, a common drawback of observation-based studies. Moreover, a lack of information regarding the intensity of IPS and TAU services use between the end of the ZhEPP trial, and follow-up interviews (i.e., from September 2014 to November 2017) in our study limits the interpretation of these findings, as some participants could have continued with IPS, while others might have dropped out. Lastly, we were unable to collect data about alternative support that participants of the TAU group may have received, which may have impacted our findings.

These drawbacks are, however, balanced by the strengths of this work. The findings of this study provide an essential perspective to the body of research on the long-term efficacy of the IPS based on the firm methodological approach and statistical analysis. Our outcome assessment was identical to the one used in the original trial, involving face-to-face interviews; thus, limiting variability in evaluation and outcome reports. To our best knowledge, this is one of the first studies to assess the effectiveness of IPS beyond 5 years of follow-up after intervention compared to conventional methods. Evaluation of the representativity of the respondent participants of the follow-up study revealed only minor differences between the groups, making the sample representative of the group of people who enrolled in the ZhEPP project. The results highlight the importance of continuous monitoring and/or intervention for people with mental disorders.

Future studies with a robust statistical approach, larger sample size, and comprehensive longitudinal follow-up are required to clarify the differences found between our study and others in the long-term impact of IPS.

## Data Availability Statement

The original contributions presented in the study are included in the article/Supplementary Material, further inquiries can be directed to the corresponding author.

## Ethics Statement

The studies involving human participants were reviewed and approved by Swiss Association of Research Ethics Committees Kantonale Ethikkommission Zürich Stampfenbachstrasse 121 8090 Zürich. Written informed consent for participation was not required for this study in accordance with the national legislation and the institutional requirements.

## Author Contributions

WK designed the follow up study and served the principal investigator. E-MP contributed to design of study. NS, LW, and SH did the statistical analysis. BW contributed to statistical analyses. E-MP drafted the manuscript. All authors participated considerably in writing of this manuscript and critically revised the final manuscript.

## Funding

This study was funded by the Swiss Federal Social Insurance Office (FSIO). It was used for scientific personnel and for the compensation of the interviews. No money was used to amplify participants‘ income.

## Conflict of Interest

The authors declare that the research was conducted in the absence of any commercial or financial relationships that could be construed as a potential conflict of interest.

## Publisher's Note

All claims expressed in this article are solely those of the authors and do not necessarily represent those of their affiliated organizations, or those of the publisher, the editors and the reviewers. Any product that may be evaluated in this article, or claim that may be made by its manufacturer, is not guaranteed or endorsed by the publisher.
