# Supplementary material for: Long-Term Effects of the Individual Placement and Support Intervention on Employment Status: 6-Year Follow-Up of a Randomized Controlled Trial
Source: Front Psychiatry. 2021 Oct 12;12:709732. doi: 10.3389/fpsyt.2021.709732 (PMC8546221; doi:10.3389/fpsyt.2021.709732)
Supplement: Supplementary file 1 [file Data_Sheet_1.docx]

# Supplementary data

**Table S1:** Logistic regression of the workplace on the first job market one year after the beginning of the study in the IPS coaching group (N = 127).

|  | | 95% CI for Odds Ratio | | |
| --- | --- | --- | --- | --- |
| Factors | β (SE) | Lower end | Odds Ratio | Upper end |
| Work history | 0.17* (0.04) | 1.09 | 1.19 | 1.29 |
| Age | 0.00 (0.02) | 0.96 | 1.00 | 1.05 |
| Female | 0.30 (0.45) | 0.56 | 1.35 | 3.23 |
| Education |  | | | |
| Primary/ elementary school |  |  |  |  |
| High school | 0.86 (0.66) | 0.64 | 2.35 | 8.61 |
| Other | 0.19 (0.70) | 0.31 | 1.21 | 4.76 |
| Medical history | 0.03 (0.03) | 0.98 | 1.03 | 1.08 |
| Diagnosis |  | | | |
| - Affective disorder |  |  |  |  |
| - Schizophrenia/ schizoaffective disorder | -0.59 (0.68) | 0.15 | 0.56 | 2.12 |
| - Personality disorder | -1.29 (0.70) | 0.07 | 0.92 | 1.09 |
| - Other | -0.09 (0.61) | 0.28 | 0.23 | 3.03 |
| Constant | -1.47 (1.14) |  | | |

CI: confidence interval; SE: standard error

R^2^ = 0.22 (Horner & Lemeshow), 0.23 (Cox & Snell), 0.31 (Nagelkerke)
*p<0.001

**Table S2:** Comparison of follow-up and drop-out sample

|  | Follow-up  n=114 | Non-respondents  n=134 | p-value | Effect size |
| --- | --- | --- | --- | --- |
| Women^b^ | 62 (54.4%) | 69 (51.5%) | 0.743 | -.029 |
| Age, yrs^a^ (*M±SD*) | 43.75 *±*10.24 | 41.31 *±*11.36 | 0.0120 | -0.225 |
| Number of years between first contact with psychological care and begin of study^a^ (*M±SD*) | 11.03 *±*8.61 | 10.10 *±*8.29 | 0.935 | -0.11 |
| Clinical diagnosis^b^ |  |  | 0.018* | 0.206 |
| Affective disorder^b^ | 66 (58.9%) | 50 (40%) | 0.005** | 0.189 |
| Schizophrenia, schizoaffective disorder^b^ | 11 (9.8%) | 23 (18.4%) | 0.090 | -0.122 |
| Personality disorder^b^ | 15 (13.4%) | 28 (22.4%) | 0.104 | -0.117 |
| Other^b^ | 20 (44.7%) | 24 (19.2%) | 0.922 | -0.017 |
| Comorbidities (yes)^b^ | 51 (44.7%) | 59 (44%) | 1.000 | 0.007 |
| Hospitalizations |  |  | 0.901 | 0.052 |
| None | 33 (29.5%) | 39 (30%) | 0.928 | 0.006 |
| 1-5 | 69 (61.6%) | 76 (58.5%) | 0.619 | 0.032 |
| 6-10 | 8 (7.1%) | 11 (8.5%) | 0.704 | 0.024 |
| 11+ | 2 (1.8%) | 4 (3.1%) | 0.818 | 0.041 |
| Highest Level of Education^b^ |  |  | 0.143 | 0.163 |
| Primary School^b^ | 73 (64.6%) | 101 (77.1%) | 0.044* | -0.138 |
| Secondary school diploma^b^ | 23 (20.4%) | 14 (10.6%) | 0.055 | 0.134 |
| Other diploma^b^ | 17 (15%) | 16 (12.1%) | 0.648 | 0.041 |
| Living situation^b^ |  |  | 0.498 | 0.110 |
| Single^b^ | 60 (53.1%) | 72 (54.1%) | 0.922 | -0.014 |
| Living with partner/married^b^ | 31 (27.4%) | 32 (24.1%) | 0.672 | 0.036 |
| Living with relatives^b^ | 10 (8.8%) | 18 (7.5%) | 0.331 | -0.075 |
| Living with others^b^ | 12 (10.6%) | 10 (7.5%) | 0.544 | 0.053 |

Abbreviations: *M*=Mean, SD=standard deviation. In relation to baseline. Cohens *d’* used for effect size for fixed variables, Cramers *V* used for effect size for categorial variables.

^a^ Mann-Whitney-U-test, ^b^ Pearson Chi-squared test.
**p<0.05, **p<0.01, ***p<0.001.*
